# Comparison between conventional and comprehensive sequencing approaches for genetic diagnosis of Alport syndrome

**DOI:** 10.1002/mgg3.883

**Published:** 2019-07-30

**Authors:** Tomohiko Yamamura, Kandai Nozu, Shogo Minamikawa, Tomoko Horinouchi, Nana Sakakibara, China Nagano, Yuya Aoto, Shinya Ishiko, Koichi Nakanishi, Yuko Shima, Hiroaki Nagase, Rini Rossanti, Ming J. Ye, Yoshimi Nozu, Shingo Ishimori, Naoya Morisada, Hiroshi Kaito, Kazumoto Iijima

**Affiliations:** ^1^ Department of Pediatrics Kobe University Graduate School of Medicine Kobe Hyogo Japan; ^2^ Department of Child Health and Welfare (Pediatrics), Graduate School of Medicine University of the Ryukyus Nishihara Okinawa Japan; ^3^ Department of Pediatrics Wakayama Medical University Wakayama Japan

**Keywords:** Alport syndrome, next‐generation sequencing, podocyte‐related gene, targeted exome sequencing

## Abstract

**Background:**

Alport syndrome (AS) is a hereditary disease caused by mutations in COL4A3‐5 genes. Recently, comprehensive genetic analysis has become the first‐line diagnostic tool for AS. However, no reports comparing mutation identification rates between conventional sequencing and comprehensive screening have been published.

**Methods:**

In this study, 441 patients clinically suspected of having AS were divided into two groups and compared. The initial mutational analysis method involved targeted exome sequencing using next‐generation sequencing (NGS) (*n* = 147, NGS group) or Sanger sequencing for *COL4A3/COL4A4/COL4A5* (*n* = 294, Sanger group).

**Results:**

In the NGS group, 126 patients (86%) were diagnosed with AS by NGS, while two had pathogenic mutations in other genes, *NPHS1* and *EYA1*. Further, 239 patients (81%) were diagnosed with AS by initial analysis in the Sanger group. Thirteen patients who were negative for mutation detection in the Sanger group were analyzed by NGS; three were diagnosed with AS. Two had mutations in *CLCN5* or *LAMB2*. The final variant detection rate was 90%.

**Discussion:**

Our results reveal that Sanger sequencing and targeted exome sequencing have high diagnostic ability. NGS also has the advantage of detecting other inherited kidney diseases and pathogenic mutations missed by Sanger sequencing.

## 1 INTRODUCTION

Alport syndrome (AS) is a hereditary disease caused by mutations in collagen type‐IV alpha chain genes (specifically, *COL4A3*/*COL4A4*/*COL4A5*, OMIM: 12007, 120131, 303630), which is characterized by progressive renal involvement, hearing loss, and ocular abnormalities (Kashtan & Michael, 1996). Mutations in *COL4A5*, encoding the collagen type IV α5 chain, are responsible for X‐linked AS (XLAS), which is usually more severe in men than women (Jais et al., 2000, 2003; Yamamura et al., 2017). Mutations in *COL4A3* and *COL4A4*, encoding the collagen type IV α3 and α4 chains, are responsible for autosomal AS. In autosomal recessive AS (ARAS), patients frequently develop end‐stage renal disease (ESRD) before the end of their second decade, with men and women similarly affected. In autosomal dominant AS (ADAS), patients usually progress to ESRD later than with ARAS. Although frequencies of these forms have been estimated to be 80% for XLAS, 15% for ARAS, and 5% for ADAS (Kashtan & Segal, 2011), recent studies have suggested that the proportion of ADAS is greater than previous estimations, especially in Southern European populations (Fallerini et al., 2014; Moriniere et al., 2014; Nabais Sa, Sampaio, et al., 2015; Nabais Sa, Storey, et al., 2015). It has also been reported that treatment with angiotensin converting enzyme inhibitors can delay development of ESRD. Accordingly, it is recommended that treatment be started immediately after diagnosis of XLAS and ARAS in males or XLAS presenting with albuminuria in females (Gross et al., 2012; Savige et al., 2013). Definitive diagnosis of AS, including mode of inheritance, is essential for starting treatment, prediction of kidney prognosis, and genetic counseling.

Previously, Sanger sequencing was widely used for genetic diagnosis of AS. However, screening of all three genes by conventional Sanger sequencing is time‐consuming and expensive because each gene contains approximately 50 exons with no mutational hotspots (Artuso et al., 2012). Recently, genetic analysis using next‐generation sequencing (NGS) has enabled comprehensive screening of many genes, with some recommending the use of this technique (Wei et al., 2011). Nonetheless, few studies involving comprehensive gene testing for AS on a large scale have been performed (Artuso et al., 2012; Fallerini et al., 2014; Moriniere et al., 2014; Wei et al., 2011), and indeed no study has compared the mutation identification rate between the conventional Sanger sequencing approach and comprehensive analysis for a large cohort. In addition, some inherited kidney diseases, such as myosin heavy chain 9 (*MYH9*)‐related nephropathy, show clinical courses and/or pathological findings similar to those of AS and can consequently be misdiagnosed (Seri et al., 2000). In general, conventional mutational screening for only the *COL4A3/COL4A4/COL4A/5* genes by Sanger sequencing cannot detect pathogenic variants in other genes, although this is possible using NGS analysis. However, no study has described cases with a clinical diagnosis of AS that were subsequently deemed to be another inherited kidney disease by a NGS approach. Hence, in this study, we compared the screening efficacy between conventional and comprehensive genetic approaches.

## 2 MATERIALS AND METHODS

### 2.1 Ethical consideration

All procedures were reviewed and approved by the Institutional Review Board of Kobe University School of Medicine. Informed consent was obtained from patients or their parents.

### 2.2 Patients

A total of 441 unrelated patients clinically suspected of having AS were analyzed in this study. They were referred to our hospital for genetic diagnosis from January 2006 to December 2017. Patients who satisfied one of the following criteria were included in this study: those with (a) hematuria and proteinuria as well as a renal pathological evaluation. Pathological findings included specific changes for AS such as diffuse thinning and/or lamellation of the glomerular basement membrane, or a specific collagen type IV α5 chain expression pattern by immunofluorescence; (b) at least hematuria and a family history of AS; or (c) at least hematuria and a family history of ESRD or chronic kidney disease (CKD) (stage II–IV) of unknown cause.

All patients were divided into the following two groups: 294 patients referred to our institute prior to November 2015 and initially analyzed by Sanger sequencing (Sanger group), and 147 patients referred to our institute after November 2015 and initially analyzed by NGS (NGS group). In addition, 13 patients from the Sanger group, in whom no pathogenic variants were detected in the *COL4A3/COL4A4/COL4A5* genes by a traditional approach, were also analyzed by NGS (COL4A3: NM_000091.4, COL4A4: NM_000092.4, COL4A5: NM_000495.4).

### 2.3 Mutational analysis

**Figure 1:**
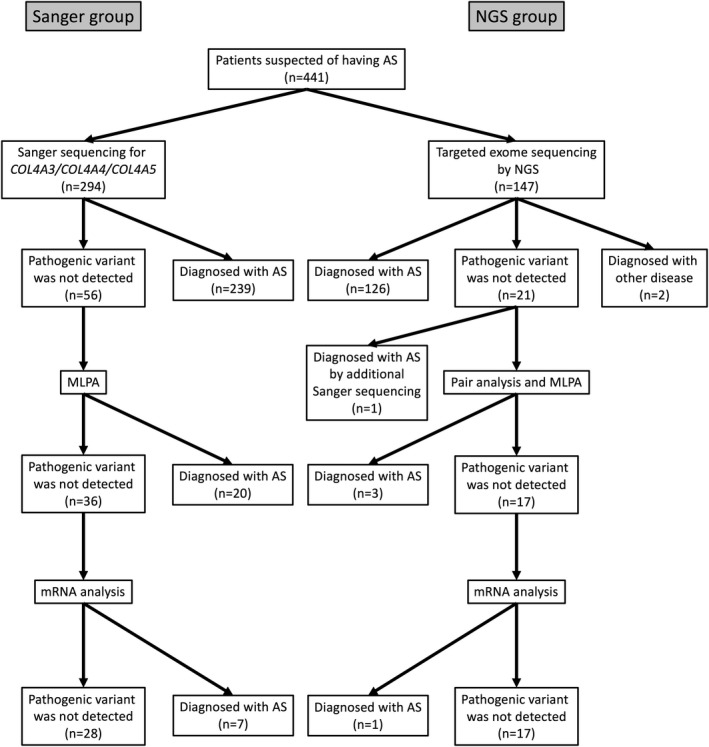
Mutational analysis approach for this study. A total of 294 patients were analyzed by Sanger sequencing (Sanger group), while 147 patients were analyzed by next‐generation sequencing (NGS; NGS group). For cases in the NGS group, if targeted exome sequencing did not detect any pathogenic variants, screening for copy number variations (CNVs) of the *COL4A4/COL4A4/COL4A5* genes was performed using pair analysis. When pair analysis detected possible CNVs in cases, multiplex ligation‐dependent probe amplification (MLPA) was used to identify them. In addition, for patients with no obvious pathogenic variants determined by the former methods, RNA sequencing using reverse transcription PCR was performed. For patients in the Sanger group, Sanger sequencing for the *COL4A3/COL4A4/COL4A5* genes was first used instead of NGS analysis, followed by MLPA and RNA sequencing for cases with no variants detected

Mutational analysis was performed in a stepwise manner, as shown in Figure 1. For cases in the NGS group, targeted exome sequencing was first performed and then screening for copy number variations (CNVs) in the *COL4A4/COL4A4/COL4A5* genes was performed using pair analysis for patients in whom no pathogenic variants were detected by targeted exome sequencing. When pair analysis detected the possibility of CNVs in cases, multiplex ligation‐dependent probe amplification (MLPA) was used to confirm CNVs. In addition, for patients with no obvious pathogenic variants, as determined by the former methods, RNA sequencing using reverse transcription polymerase chain reaction (PCR) was performed to detect aberrant splicing by intronic variants. For patients in the Sanger group, Sanger sequencing for the *COL4A3/COL4A4/COL4A5* genes was first used instead of NGS analysis, followed by MLPA and RNA sequencing for cases with no variants detected. The details of the method used for each analysis are presented below.

Novel variants were determined as pathogenic based on previous reports(Kobayashi, Kakihara, & Uchiyama, 2008; Savige et al., 2018), as follows: (a) nonsense variants; (b) exonic deletions/insertions or duplications; (c) mutations located in consensus splicing sites; (d) mutations located out with consensus splicing sites (including deep intronic regions) with abnormal mRNA shown by RNA analysis; (e) glycine missense variants in intermediate collagenous domains; and (f) missense variants in non‐collagenous domains. In particular, regarding the missense variants in (e) and (f), pathogenicity was determined based on expert guidelines for the genetic diagnosis of glycine missense variants in AS (Savige et al., 2018), and general guidelines developed in the US (American College of Medical Genetics and Genomics [ACMG] classification) for interpretation of missense sequence variants in noncollagenous domains (Richards et al., 2015).

### 2.4 Sanger sequencing

Sanger sequencing was performed for *COL4A3/COL4A4/COL4A5* by PCR and direct sequencing of genomic DNA for all exons and exon–intron boundaries. Blood samples were collected from patients and/or family members, and genomic DNA isolated from peripheral blood leukocytes using the Quick Gene Mini 80 System (Kurabo), in accordance with the manufacturer's instructions. For genomic DNA analysis, 51 exons of *COL4A5*, 52 exons of *COL4A3*, and 48 exons of *COL4A4* were amplified by PCR, as described previously (Hashimura et al., 2014). PCR‐amplified products were then purified and subjected to direct sequencing using a Dye Terminator Sequencing Kit (Amersham Biosciences) with an automatic DNA sequencer (model ABI Prism 3130; PerkinElmer). When pathogenic variants were identified in one gene, other genes were not examined, which means that digenic variants may have been missed in the Sanger group.

### 2.5 Targeted exome sequencing

A custom panel was designed for targeted sequences (the gene list is shown in Table S1). NGS samples were prepared using a HaloPlex Target Enrichment System Kit (Agilent Technologies) to capture 45 genes, in accordance with the manufacturer's instructions. Amplified target libraries were sequenced using MiSeq (Illumina) and analyzed with SureCall (v.3.0; Agilent Technologies). Detected variants were confirmed by Sanger sequencing.

In addition, to screen CNVs of the *COL4A3/COL4A4/COL4A5* genes for the NGS group, analyzed NGS data were used for pair analysis using SureCall software. In brief, pair analysis compared NGS data from patients with suspected CNVs to a reference lacking CNVs, as previously described (Nagano et al., 2018). All CNVs detected by pair analysis were confirmed by MLPA.

### 2.6 MLPA analysis and pair analysis

MLPA was performed using SALSA P191/192 for *COL4A5*, P439 for *COL4A3*, and P444 for *COL4A4*, as recommended by the manufacturer (MRC‐Holland). Briefly, 50–100 ng of genomic DNA in 5 µl of deionized water was denatured and hybridized overnight with probe mix. Ligation was performed using the SALSA Ligase 65 enzyme, with PCR amplification performed using SALSA PCR Primer Mix. Amplification products and Size Standard 600 (Thermo Fisher) were mixed thoroughly and subjected to capillary electrophoresis on Gene Mapper v.3.7 (Thermo Fisher).

### 2.7 RNA sequencing

Total RNA was extracted from blood leukocytes and/or urinary sediments. RNA from leukocytes was isolated using RNA*later* RNA Stabilization Reagent and RNA Blood Mini Kit (Qiagen Inc.), and reverse transcribed into cDNA using random hexamers and Superscript III Kit (Invitrogen), as previously described (Nozu et al., 2014). RNA from urinary sediments was isolated using ZR Urine RNA Isolation Kit (Zymo Research) according to the manufacturer's instructions, and reverse transcribed into cDNA as described. However, cDNA was amplified by nested PCR using primer pairs for *COL4A3*/*COL4A4*/*COL4A5*, as described previously albeit with slight modifications (primer designs can be provided on request) (Inoue et al., 1999). PCR‐amplified products were purified and subjected to Sanger sequencing. After detecting abnormal splicing by cDNA analysis, genome DNA variants causing aberrant splicing were confirmed by Sanger sequencing.

## 3 RESULTS

### 3.1 Summary of mutational analysis

Regarding the 294 patients in the Sanger group, 239 (81%) were genetically diagnosed with AS by Sanger sequencing (*COL4A5*: 187, 78%; *COL4A3*: 24, 10%; *COL4A4*: 28, 12%). Further, 20 patients were diagnosed with AS and CNVs (*COL4A5*: 18; *COL4A3*: 1; *COL4A4*: 1) by MLPA analysis, while 7 patients were shown to have cryptic exon insertions by deep intronic single‐base substitutions using RNA sequencing. In contrast, of the 147 patients in the NGS group, 126 (86%) were genetically diagnosed with AS by NGS (*COL4A5*: 79, 63%; *COL4A3*: 22, 17%; *COL4A4*: 25, 20%). Three patients were shown to have CNVs in the *COL4A5* gene by pair analysis, which were confirmed by MLPA analysis. One patient was shown to have a cryptic exon insertion by deep intronic single‐base substitution using RNA sequencing. In addition, one patient was shown to have a single‐base duplication in exon 10 of the *COL4A5* gene by Sanger sequencing. This finding was obtained because NGS analysis showed no amplification of exon 10 of the *COL4A5* gene, therefore we performed additional Sanger sequencing for only this exon for cases in the NGS group (Figure 1, Table 1).

**Table 1:** Summary of genetic analyses for all 441 patients

Characteristics	Number of patients	
	Targeted exome sequencing	Sanger sequencing
Patients diagnosed with AS by initial sequencing	126	239
Patients diagnosed with AS by MLPA analysis	3	20
Patients diagnosed with AS by cDNA analysis	1	7
Patients diagnosed with AS by additional Sanger sequencing	1	‐
Patients with *COL4A5* mutation	84	211
Patients with *COL4A3* mutation	22	26
Patients with *COL4A4* mutation	25	29
Patients diagnosed with other diseases by NGS	2^a^	‐
No mutations were detected	14	28
Total	147	294

Note

Abbreviations: AS, Alport syndrome; MLPA, multiplex ligation‐dependent probe amplification; NGS, next generation sequence

a Compound heterozygous *NPHS1* mutation and heterozygous *EYA1* mutation

Mutation identification rates were similar between both groups (81% vs. 86%, *p* = .220, χ^2^ analysis). In addition, NGS analysis revealed that one patient had a compound heterozygous *NPHS1* gene mutation, while another had an *EYA1* gene mutation. These patients were re‐diagnosed with nephrotic syndrome and brachio‐otorenal syndrome, respectively. The patient with the *NPHS1* mutation was referred to our institute for genetic analysis at the age of 15 because of a small amount of microscopic hematuria, moderate proteinuria, and a diffuse thinning of the glomerular basement membrane observed by kidney biopsy (matching criterion [i]). The patient with the *EYA1* mutation was referred to our institute because of hematuria, hearing loss, and a positive family history of CKD (matching criterion [iii]). He did not have an ear malformation or branchial cleft fistulae, however, his father had auricular fistulae with the same *EYA1* mutation detected.

In addition, we calculated variant detection rates separately for each inclusion criteria, and obtained rates of 91% (*n* = 390) in patients included in this study by criterion (i), 100% (*n* = 16) in patients with criterion (ii) but not with criterion (i), and 75% (*n* = 35) in patients with criterion (iii) but not with criterion (i) or (ii).

### 3.2 Mode of inheritance

Regarding the mode of inheritance, patients with XLAS were most common (*n* = 295, 74%), followed by those with ADAS (*n* = 67, 17%). Patients with ARAS were relatively rare (*n* = 35, 9%) (Table 2). All patients diagnosed with ADAS in this study satisfied one of the inclusion criteria, and had a heterozygous mutation in *COL4A3* or *COL4A4*. Among all 67 patients with ADAS, 63 patients underwent renal biopsy (fulfilled criterion [i]), one patient fulfilled criterion (ii) but not criterion (i), while three patients fulfilled criterion (iii) but not criterion (i) or (ii).

**Table 2:** Mutation types in each gene

Mutation features	*COL4A3*	*COL4A4*	*COL4A5*
Mutations	45	48	249
Mutated alleles	70	67	295
Patients	48	54	295
Patients with hemizygous mutation	0	0	157
Patients with homozygous mutation	4	1	0
Patients with compound heterozygous mutation	18	12	0
Patients with heterozygous mutation	26	41	138
Missense mutations	43	43	153
Nonsense mutations	4	4	25
Splicing mutations	7	4	47
Small rearrangements (deletions/insertions/duplications)	11	14	49
Large rearrangements	1	1	21

Regarding gene types, we identified 342 different variants considered to be disease‐causing on 432 alleles in 397 unrelated families among 441 families, suggesting none of the three genes contained mutational hotspots. *COL4A5* mutations were most common (*n* = 249), while the number of *COL4A3* and *COL4A4* mutations were almost the same (*n* = 45 and 48, respectively). The mutational features of each gene are described in Table 2.

In total, 441 families were included in this study. Among them, 25 families with ADAS (Kamiyoshi et al., 2016), 24 families with ARAS (Oka et al., 2014), and 215 families with XLAS (Hashimura et al., 2014) (Yamamura et al., 2017) (Horinouchi et al., 2018) have already been reported by our group.

### 3.3 Analysis cost

The cost of Sanger sequencing was approximately US$230–250 for all of the analyzed *COL4A* genes, with the *COL4A5* gene usually screened first. If screening for all exons of *COL4A5* failed to detect any pathogenic variants, the cost doubled or tripled depending on the number of screened *COL4A3* and *COL4A4* genes. In contrast, the cost of targeted exome sequencing using NGS was approximately US$230 for one sample.

### 3.4 Additional analysis for undiagnosed patients

For 13 undiagnosed patients in the Sanger group, we performed NGS analysis, and found that three patients had AS with mutations that had been missed by Sanger sequencing. This was because the Sanger sequencing chromatogram showed a complete match between variant and wild‐type peaks in a heterozygous state (Figure S1). In addition, two patients were shown to have other gene mutations in specifically, chloride voltage‐gated channel 5 (*CLCN5*) and laminin subunit beta 2 (*LAMB2*) (Table 3). The patient with the *CLCN5* mutation was referred to our institute for genetic analysis because of hematuria, mild proteinuria, mild hearing loss, and diffuse thinning of the glomerular basement membrane (matching criterion [i]). The patient with the *LAMB2* mutation was referred to our institute because of hematuria, proteinuria, and glomerular basement membrane lamellation (matching criterion [i]).

**Table 3:** Summary of NGS analysis for patients in the Sanger group with no variants detected

Characteristics	Number of patients
Patients diagnosed with AS by NGS	3
Patients with *COL4A5* mutation	2
Patients with *COL4A3* mutation	1
Patients diagnosed with other diseases by NGS	2^a^
No mutations were detected	8
Total	13

Note

Abbreviations: AS, Alport syndrome; NGS, next generation sequence

a Hemizygous *CLCN5* gene mutation and compound heterozygous *LAMB2* gene mutation

## 4 DISCUSSION

Here, we have compared a diagnostic strategy using conventional Sanger sequencing and comprehensive targeted exome sequencing by NGS for patients suspected of having AS based on their clinical and/or pathological findings. Our results show high mutation detection rates in both groups.

Pathogenic mutations in the *COL4A3*, *COL4A4*, and *COL4A5* genes resulted in a spectrum of thin basement membrane disease or AS. Accurate diagnosis of AS, including clarifying the mode of inheritance, is important for providing appropriate genetic counseling and correctly predicting prognosis. Regarding patients with AS, there is a major difference in renal prognosis among the different modes of inheritance, with a strong genotype–phenotype correlation observed in male XLAS patients (Bekheirnia et al., 2010; Gross, Netzer, Lambrecht, Seibold, & Weber, 2002; Jais et al., 2000; Kamiyoshi et al., 2016; Oka et al., 2014). Careful clinical evaluation including histopathology, analysis of collagen type IV expression in glomerular basement membrane or skin, and pedigree information is usually sufficient to estimate mode of inheritance. However, genetic analysis is more accurate for determining mode of inheritance, especially in sporadic cases.

Previously, Sanger sequencing was widely used for genetic diagnosis of AS. However, screening of the *COL4A3/COL4A4/COL4A5* genes by conventional Sanger sequencing is time‐consuming, labor‐intensive, and expensive. Reasons for this involve the extremely large numbers of exons that these genes contain (52, 48, and 51, respectively) (Artuso et al., 2012), and also their lack of mutational hotspots. To date, more than 950 pathogenic variants of the *COL4A5* gene have been reported in the Human Gene Mutation Database database (http://www.ghmd.cf.ac.uk/), yet actually the majority of mutations detected by our analysis were novel. Therefore, we had to check all the exons of the target *COL4A* gene. It is often necessary to analyze all three genes (i.e., *COL4A3/COL4A4/COL4A5*) for patients suspected of having AS because the mode of inheritance cannot be estimated from clinical findings. However, when pathogenic variants are detected by the Sanger screening method, there is no need to examine further genes, which can lead to missing of digenic gene variants in other *COL4A* genes.

The use of NGS analysis has spread rapidly in recent years and might resolve these problems. However, only a few reports have provided detailed NGS analysis of AS (Artuso et al., 2012; Fallerini et al., 2014; Kovacs et al., 2016; Moriniere et al., 2014). In addition, no reports have compared Sanger and NGS screening results. Moreover, no reports have described clinically suspected AS cases that were shown to be different inherited kidney diseases by comprehensive NGS analysis. In our study, various benefits of a comprehensive diagnostic approach using NGS for the diagnosis of AS were revealed.

First, targeted exome sequencing using NGS has high diagnostic ability. This study revealed that at our institute, conventional Sanger sequencing identified pathogenic variants in 81% of patients clinically diagnosed with AS. In contrast, comprehensive targeted exome sequencing using NGS identified pathogenic variants in 86% of patients. A previous study also found that screening of the *COL4A34/COL4A4/COL4A5* genes using NGS identified mutations in 82% of 101 cases (Moriniere et al., 2014). Altogether, these findings show that a comprehensive approach using targeted exome sequencing has high diagnostic ability.

Second, NGS analysis can diagnose other inherited kidney diseases that show clinical courses and/or pathological findings similar to those of AS. In fact, pathogenic variants in various genes responsible for inherited kidney diseases (patients with *NPHS1*, *EYA1*, *LAMB2*, and *CLCN5* gene mutations) were detected by NGS in our study.

Third, in this study, we reduced the cost of mutational analysis by targeted sequencing to one‐third that for analyzing all exons of the *COL4A3/COL4A4/COL4A5* genes by Sanger sequencing.

In addition, comprehensive screening using NGS detected digenic mutations in AS. In recent years, it has been reported that podocyte‐related genes such as podocin (*NPHS2*) and myosin IE (*MYO1E*) might act as modifiers of AS (Lennon et al., 2015; Tonna et al., 2008). Mencarelli *et al*., also showed that digenic variants in any two genes among *COL4A3*, *COL4A4*, and *COL4A5* cause more severe phenotypes than monogenic variants of one of these genes (Mencarelli et al., 2015). In fact, using NGS we detected two ADAS cases with digenic variants of both *COL4A3* and *COL4A4* (Kamiyoshi et al., 2016). Therefore, it may be worth searching for modifier genes in patients with phenotypes that are more severe than expected from their modes of inheritance or genotypes.

Among 13 cases with negative results for mutation detection in the Sanger group, 3 cases were newly diagnosed with AS. After obtaining our NGS results, we analyzed the chromatograms obtained by Sanger sequencing for confirmation and found mutations that had been missed because the heterozygote variant peaks matched wild‐type peaks (Figure S1). However, upon closer inspection, they were easy to detect. Although confirmation of sequencing results is routinely performed using both software and manually at our institute, this shows that some variants can be missed by human error. In contrast, NGS analytical software usually correctly picks up pathogenic variants.

NGS analysis did not identify specific types of variants. Indeed, it is difficult to detect CNVs by NGS analysis because the number of reads does not accurately reflect the amount of genomic DNA in the target region. In addition, deep intronic variants cannot be detected by targeted exome sequence because only exons and their flanking intronic regions are read by this system. Thus, it is necessary to include a CNV search or RNA sequencing to detect splicing variants for cases in which initial NGS analysis does not identify any pathogenic variants. Nonetheless, we have already established a screening strategy for CNVs in various inherited kidney diseases using pair analysis (Nagano et al., 2018), for which NGS analysis successfully detected CNVs in three AS cases.

Targeted exome sequencing might lead to overlooking of pathogenic variants. NGS metrics in our cohort showed greater than 250‐fold mean coverage with 97% of the region of interest covered by at least 20 reads in almost all cases. Nevertheless, one patient had a pathogenic mutation in an unreadable exonic region. In this study, we designed a custom panel for all exons of targeted genes. However, probes for amplifying exon 10 of the *COL4A5* gene could not be designed due to problems in combining restriction enzymes that do not match the relevant sequence. Therefore, we checked coverage of NGS analysis and included additional Sanger sequencing for unreadable exonic regions in patients in whom targeted exome sequencing did not detect any pathogenic variants. Despite this, the additional analysis involved only a small expense and takes less time than Sanger sequencing because the unreadable region is quite short.

In addition, our results provide novel findings on the proportion of each mode of inheritance in the Japanese population. Although the percentage of patients who were genetically diagnosed with AS did not differ between the conventional Sanger sequencing approach and targeted exome sequencing approach, the proportion of patients with *COL4A5* gene mutations was substantially lower (79% and 64%), while the proportion of patients with *COL4A3* or *COL4A4* variants was correspondingly higher in the NGS group. A higher detection rate in *COL4A3* or *COL4A4* in the NGS group was similar to recent reports on Southern European populations (Fallerini et al., 2014; Nabais Sa, Sampaio, et al., 2015; Nabais Sa, Storey, et al., 2015). The reason for the higher detection rate in autosomal genes might be due to the inheritance mode of ADAS, which has recently been widely recognized among nephrologists in Japan because of several studies suggesting that the prevalence of ADAS is higher than assumed, not only in Southern European populations, but also in the Japanese population (Fallerini et al., 2014; Kamiyoshi et al., 2016; Nabais Sa, Sampaio, et al., 2015; Nabais Sa, Storey, et al., 2015).

In conclusion, our study shows that both Sanger sequencing and targeted exome sequencing have high diagnostic ability for patients clinically suspected of having AS. In addition, the overall cost‐effectiveness of targeted exome sequencing was similar to Sanger sequencing when three *COL4A3–5* genes were analyzed. Moreover, NGS analysis showed that some clinically diagnosed AS patients have other inherited kidney diseases.

## CONFLICT OF INTEREST

Kandai Nozu and Kazumoto Iijima have filed a patent application on the development of antisense nucleotides for exon skipping therapy in Alport syndrome. Kandai Nozu has received lecture fees from Novartis Pharmaceuticals Corporation fees and consulting fees from Kyowa Hakko Kirin Co., Ltd. Kazumoto Iijima has received a grant support from Daiichi Sankyo Co., Ltd., consulting fees from Takeda Pharmaceutical Company and Kyowa Hakko Kirin Co., Ltd., and lecture fees from Chugai Pharmaceutical Co., Ltd., Takeda Pharmaceutical Company and Kyowa Hakko Kirin Co., Ltd.
