# Supplementary material for: Comparison between conventional and comprehensive sequencing approaches for genetic diagnosis of Alport syndrome
Source: Mol Genet Genomic Med. 2019 Jul 30;7(9):e883. doi: 10.1002/mgg3.883 (PMC6732293; doi:10.1002/mgg3.883)
Supplement: Supplementary file 1 [file MGG3-7-e883-s001.docx]

**Supplementary Table 1. Targeted genes in the NGS custom panel**

| Gene | | Gene | | Gene | |
| --- | --- | --- | --- | --- | --- |
| 1 | *ACTN4* | 16 | *FAT1* | 31 | *NUP107* |
| 2 | *ADCK4* | 17 | *GLEPP1* | 32 | *NUP205* |
| 3 | *ANLN* | 18 | *HNF1B* | 33 | *NUP93* |
| 4 | *ARHGAP24* | 19 | *INF2* | 34 | *PAX2* |
| 5 | *ARHGDIA* | 20 | *ITGA3* | 35 | *PDSS2* |
| 6 | *CD2AP* | 21 | *ITGB4* | 36 | *PLCE1* |
| 7 | *COL4A3* | 22 | *KANK1* | 37 | *PTPRO* |
| 8 | *COL4A4* | 23 | *KANK2* | 38 | *SCARB2* |
| 9 | *COL4A5* | 24 | *KANK4* | 39 | *SMARCAL1* |
| 10 | *COQ2* | 25 | *LAMB2* | 40 | *TRPC6* |
| 11 | *COQ6* | 26 | *LMX1B* | 41 | *TTC21B* |
| 12 | *CRB2* | 27 | *MYH9* | 42 | *UMOD* |
| 13 | *CUBN* | 28 | *MYO1E* | 43 | *WDR73* |
| 14 | *DGKE* | 29 | *NPHS1* | 44 | *WT1* |
| 15 | *EMP2* | 30 | *NPHS2* | 45 | *XPO5* |

NGS, next-generation sequencing.

**Supplementary Figure 1. Mutational analysis results of a case in which Sanger sequencing overlooked the pathogenic variant.**

A: Left, Sanger sequencing of exon 36 in the *COL4A5* gene of a patient in which the variant was missed by both software and manual examination. Further inspection of the Sanger sequencing chromatogram showed that the variant (C) and normal nucleotide (T) waves were the same shape. This variant was not recognized by a software check. Right, Sanger sequencing of exon 34 in the *COL4A4* gene of another patient. This variant was recognized by a software check. B: Next-generation sequencing analysis clearly revealed the variant nucleotide.
